# Association of serum selenium levels with inflammatory and oxidative stress markers in patients with post-infarction heart failure: an exploratory case-control study supported by *in vitro* mechanistic evidence

**DOI:** 10.3389/fcvm.2026.1821567

**Published:** 2026-06-09

**Authors:** Fabrice Yves Ndjana Lessomo, Ruiyang Zhu, Jingjing Wan, Jinbo Zhao, Yuanhong Li, Ye Mao, Liping Guo

**Affiliations:** 1 Clinical Nutrition Department, Central Hospital of Tujia and Miao Autonomous Prefecture, Enshi, China; 2 Department of Cardiology, Zhongnan Hospital, Wuhan University, Wuhan, China; 3 Center for Gene Diagnosis, and Department of Clinical Laboratory Medicine, Zhongnan Hospital of Wuhan University, Wuhan, China; 4 Cardiovascular Disease Center, Central Hospital of Tujia and Miao Autonomous Prefecture, Enshi, China

**Keywords:** antioxidant, cardiomyocytes, case-control study, cytokines, fibrosis, heart failure, inflammation, myocardial infarction

## Abstract

**Background:**

As a trace element, Selenium is essential for antioxidant defense and immune regulation; however, its influence on post-infarction heart failure (HF) remains underexplored.

**Methods:**

The present study used a hybrid approach, combining a retrospective case-control analysis with *in vitro* mechanistic assays. Serum Selenium, inflammatory cytokines (TNF-α, IL-6), oxidative stress markers (MDA, SOD), and left ventricular ejection fraction (LVEF) were assessed in 46 patients with post-infarction HF and 43 healthy controls. In parallel, H9C2 cardiomyocytes exposed to either lipopolysaccharide (LPS) or palmitic acid (PA) were assessed for inflammatory and fibrotic gene expression, with and without Selenium treatment.

**Results:**

HF subjects had significantly lower Selenium and SOD levels than controls (all *p* < 0.05), and increased TNF-α, IL-6, and MDA. Selenium as an anti-inflammatory element was positively associated with LVEF and negatively associated with TNF-α, IL-6, and MDA. *In vitro*, Selenium was associated with decreased expression of pro-inflammatory (TNF-α, IL-1β and IL-6) and pro-fibrotic (Collagen I, III, and *α*-SMA) genes during stress. Exploratory subgroup analysis suggested heterogeneous distributions of Selenium and oxidative stress markers across HF phenotypes, with the lowest Selenium levels observed in the HFmrEF subgroup. *In vitro*, Selenium reduced stress-induced expression of pro-inflammatory (TNF-a, IL-1b) and pro-fibrotic (Collagen I, Collagen III, a-SMA) genes.

**Conclusion:**

Selenium deficiency was associated with greater inflammatory and oxidative stress burden in post-infarction HF. *In vitro* findings indicates that Selenium may contribute to cardiac injury via inflammation and fibrotic signaling. These data support serum Selenium as an exploratory biomarker for further study in HF and provide a rationale for future longitudinal and interventional research.

## Highlights

- Selenium deficiency is associated with increased inflammation (IL-6, TNF-α) and oxidative stress (MDA) in post-infarction heart failure patients
- Selenium positively correlates with antioxidant capacity (SOD) and cardiac function (LVEF), supporting its cardioprotective role
- *In vitro* evidence demonstrates that Selenium attenuates LPS- and PA-induced pro-inflammatory and pro-fibrotic gene expression in cardiomyocytes
- Heterogeneous Selenium distribution across HF phenotypes (HFpEF, HFmrEF, HFrEF) suggests stage-dependent redox dynamics in disease progression

## 1 Introduction

Heart failure (HF) is a complex clinical syndrome caused by structural or functional abnormalities of the heart that impair its ability to meet the metabolic needs of the body. It continues to be a leading caused of worldwide disease and mortality, placing excessive stress on healthcare systems. HF remains a major cause of morbidity and mortality worldwide, and its prevalence is expected to continue rising as populations age and survival after myocardial infarction improve s(1). Some medical therapies and device therapy have improved, but the death rate at 5 years is still greater than 50% (2). It has been emphasized that true prevention or HF control is engrafted on the improvements of preventive methods through research and treatment optimization for underlying causes. Oxidative stress, mitochondrial dysfunction, neurohormonal activation, and chronic inflammation all contribute to the pathophysiology of HF (3, 4). In recent years, growing attention has been given to trace elements such as iron, zinc, and selenium because of their roles in redox balance, immune regulation, and myocardial energy metabolism (5).

Selenium (Selenium), a key trace element, has been linked to cardiovascular disease by studies on Keshan disease, an endemic cardiomyopathy prevalent in China's Selenium-deficient areas. As early as 1985 Selenium deficiency was established as a core etiological factor for the disease (6). Since then, subsequent epidemiologic and experimental studies have also linked Selenium status to coronary artery disease, myocardial infarction, and HF (7–9). Thus, pointing to the cardioprotective role of Selenium.

.Selenium is an important component of selenoproteins enzymes, such as glutathione peroxidase and thioredoxin reductase, which play a key role in reducing reactive oxygen species (ROS) and oxidative stress mediated cardiac damage. Recent research on heart failure (HF) have been oriented towards Selenium's ability to reduce inflammation. Inflammatory mediators such as TNF-a and IL-6 are increasingly recognized as drivers of cardiac remodeling and fibrosis in HF. Because Selenium may influence both redox homeostasis and inflammatory signaling, it is relevant to examine whether lower Selenium status is associated with inflammatory and oxidative stress burden in patients with post-infarction HF.

Accordingly, this study aimed to evaluate the association between serum Selenium levels and clinical biomarkers of inflammation and oxidative stress by using data from Enshi Tujia and Miao Autonomous Prefecture Central Hospital to build a retrospective case-control design, while using *in vitro* experiments to explore whether Selenium may modulate inflammatory and fibrotic responses under stress conditions. We hypothesized that Selenium deficiency contributes to the development and progression of HF by activating inflammatory cytokines and oxidative stress pathways.

## 2 Methods

### 2.1 Study design and data source

This study used a retrospective case-control design combined with H9C2 (CL0089) cell-based experimental analysis. Clinical data were obtained from the cardiology inpatient electronic medical record system and the health examination center of Enshi Central Hospital of Tujia and Miao Autonomous Prefecture between December 1 and December 31, 2021. The protocol was approved by the local ethics committee.

### 2.2 Study population and data collection and measurement

Case Group: We retrospectively screened patients hospitalized in the cardiology department who were diagnosed with heart failure secondary to prior myocardial infarction (post-infarction HF); all included patients were under follow-up after percutaneous coronary intervention (PCI). HF diagnosis was made according to the New York Heart Association (NYHA) functional classification standards. Inclusion criteria were: (1) age between 30 and 80 years; (2) heart failure duration of at least 3 months; (3) complete laboratory and echocardiographic examination data in medical records. Exclusion criteria included: (1) Severe liver or kidney dysfunction; (2) malignant tumors; (3) infection history within the past month; (4) autoimmune diseases; (5) current use of antioxidants or Selenium supplements. All patients in the case group were on consistent standard post-infarction HF medication (e.g., ACEI/ARB, β-blockers, statins). 46 patients with heart failure met the inclusion criteria and were enrolled in the study.

**Figure 1:**
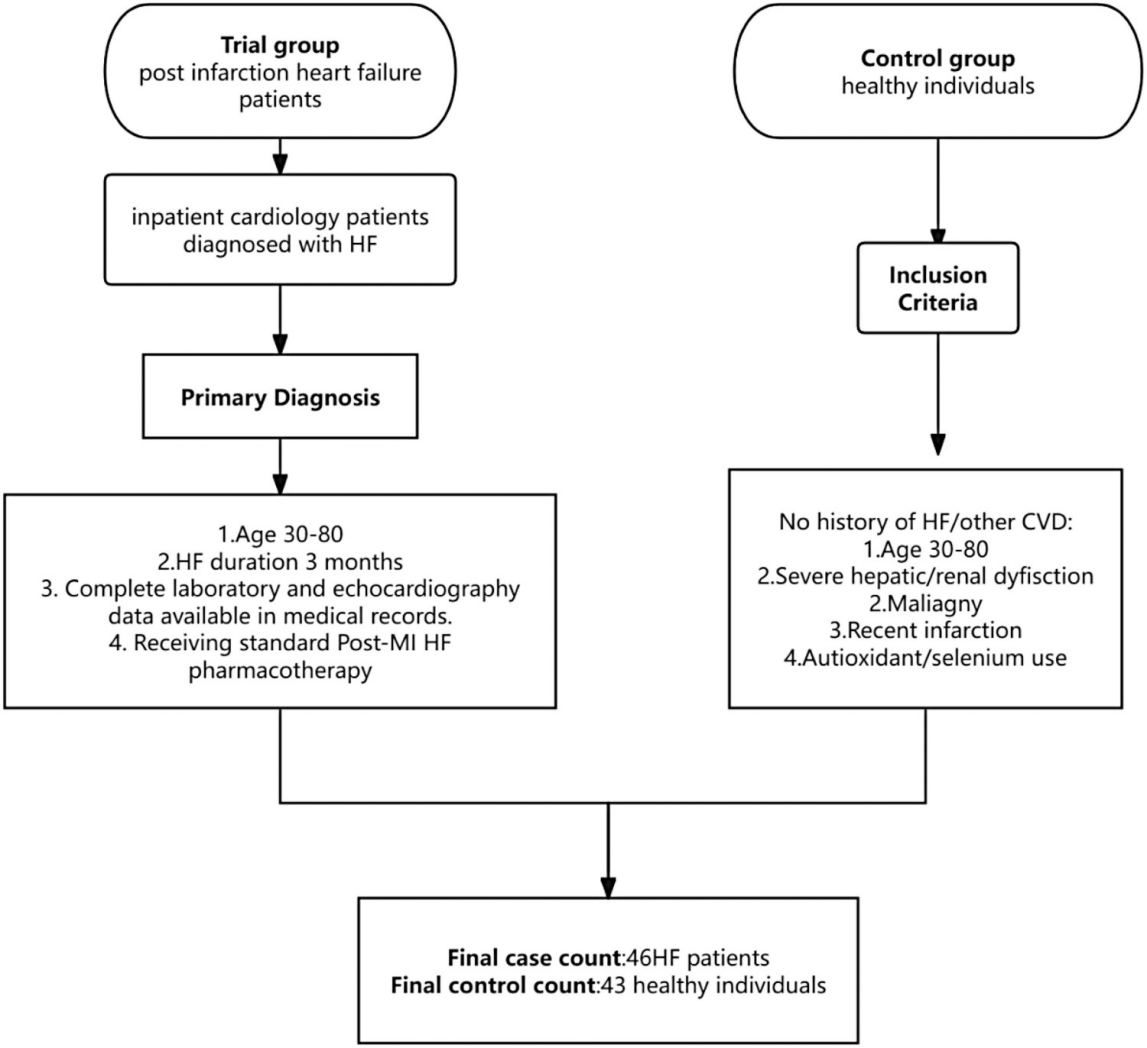
Case selection flow. Flow diagram of participant selection for the clinical case−control study. Patients with post−infarction heart failure were retrospectively identified from the cardiology inpatient database according to the predefined inclusion and exclusion criteria. Healthy controls were Selected from individuals undergoing routine health examinations during the same period. The final study population included 46 patients with post−infarction heart failure and 43 healthy controls. HF, heart failure; CVD, cardiovascular disease; MI, myocardial infarction.

Control Group: The control group consisted of healthy individuals who underwent routine health examinations at the hospital's health examination center during the same period all control participants underwent a standardized health screening protocol, including a detailed medical history and cardiovascular symptom questionnaire covering chest pain or discomfort, dyspnea, palpitations, and related symptoms; a physical examination focused on signs of heart failure or ischemic heart disease; a 12-lead resting electrocardiogram reviewed by a cardiologist for pathological Q waves, ST-segment abnormalities, or other findings suggestive of prior myocardial infarction; transthoracic echocardiography performed by experienced sonographers to assess regional wall motion abnormalities, left ventricular systolic function, and structural abnormalities; and measurement of high-sensitivity cardiac troponin I as part of the routine health examination panel. Records were independently reviewed by a second cardiologist to confirm eligibility. Inclusion criteria for the control group were: (1) age between 30 and 80 years; (2) no history of heart failure or other cardiovascular diseases and high-risk factors such as CVD family history. Exclusion criteria were the same as those for the case group, to ensure the control group was free of confounding conditions that could affect inflammatory or oxidative stress markers. A total of 43 healthy individuals were included in the control group. The control group reported no regular use of selenium (Figure 1).

All blood samples were fasting venous blood (2 mL) collected from the elbow early in the morning during patient admission or health examination. Serum was separated by centrifugation at 2000rpm for 15 min and stored at −80 °C. Plasma aliquots (1 mL) were frozen at −20 °C for transportation. Serum levels of Interleukin-6 (IL-6), tumour necrosis factor-alpha (TNF-α), superoxide dismutase (SOD), and malondialdehyde (MDA) were determined using an enzyme-linked immunosorbent assay kit (Cloud-clone corp, SELENIUMA079Hu, SELENIUMA133Hu, SELENIUMS134Hu, CEA597Ge, respectively). Plasma Selenium levels were measured using an atomic emission photoelectric optical emission spectrometer (AFS-930, Beijing) within one week of blood collection. Echocardiographic results for heart failure patients were obtained from cardiology ultrasonography examination records. These results included the left ventricular ejection fraction (LVEF), the left ventricular end-diastolic diameter (LVEDD), and the left ventricular end-systolic diameter (LVESD). All ultrasound tests were performed by skilled sonographers who adhered to established procedures.

### 2.3 Cell culture and treatment

H9C2 cardiomyocytes (CL-0089) were cultured in Dulbecco's Modified Eagle Medium (DMEM)/F12 (Gibco, USA) supplemented with 10% fetal bovine Serum (FBS; Gibco) and 1% penicillin/streptomycin in a humidified incubator at 37  °C with 5% CO_2_. Cells were stimulated with lipopolysaccharide (LPS, 1 ug/mL) or palmitic acid (PA, 200 uM) for 24 h to model inflammatory and fibrotic stress. The LPS used in this study was derived from Escherichia coli O111:B4 (Sigma-Aldrich, USA). To investigate the protective effects of Selenium, cells were pre-treated or co-treated with Sodium Selenium (Na2SeO3) at indicated concentrations (100 µM, 200 µM, or 300 µM). Cell viability was assessed using the Cell Counting Kit-8 (CCK-8) assay to determine the optimal non-toxic concentration of Selenium for subsequent experiments. To investigate the protective effects of Selenium, cells were pre-treated. All *in vitro* experiments were performed in at least three independent biological replicates (*n* = 6).

### 2.4 RNA extraction and qPCR

Total RNA was extracted from H9C2 cells using TRIzol reagent (Invitrogen, USA) according to the manufacturer's instructions. The concentration and purity of RNA were determined using a NanoDrop 2000 spectrophotometer (Thermo Fisher Scientific, USA). Subsequently, 1 µg of total RNA was reverse transcribed into cDNA using a High-Capacity cDNA Reverse Transcription Kit (Takara, Japan). Real-time quantitative PCR (RT-qPCR) was performed using the TB Green® Premix Ex Taq™ II (Takara) on a Light Cycler® 480 System (Roche, Switzerland). The amplification conditions were as follows: initial denaturation at 95 °C for 30 s, followed by 40 cycles of 95 °C for 5 s and 60 °C for 30 s. The relative mRNA expression levels of inflammatory cytokines (TNF-α, IL-1β, IL-6) and fibrosis markers (Collagen I, Collagen III, *α*-SMA) were calculated using the 2^−*ΔΔ*Ct^ method, with *GAPDH* as the internal reference. The specific primer sequences used in this study are listed in Supplementary Table S1.

### 2.5 Statistical analysis

Normal distribution for continuous variables was determined using the Shapiro–Wilk test. Normally distributed data are presented as mean ± standard deviation (SD) and compared using the independent Student's t-test. Non-normally distributed variables are expressed as median [interquartile range, IQR] and analyzed with the Mann–Whitney U test. Categorical variables are presented as frequencies (percentages) and compared using the Chi-square (*χ*^2^) test. To assess the association between Selenium and heart failure markers while accounting for potential confounders, Spearman's rank correlation analysis was performed. Given the demographic differences and the significant age difference between groups, multivariable logistic regression analysis was performed to determine if Selenium levels were independently associated with the presence of HF after adjusting for age and gender. Additionally, multivariable linear regression was used to assess the relationship between Selenium and inflammatory markers, controlling for age as a confounding factor. A two-tailed *p*-value < 0.05 was considered statistically significant. All analyses were performed using SPSS 26.0 and GraphPad Prism 9.0.

## 3 Results

### 3.1 Case selection and baseline characteristics

A total of 89 participants were included in the study (46 post-infarction HF patients and 43 healthy controls). The baseline characteristics are summarized in Table 1. The HF group exhibited significantly lower serum Selenium levels compared to the control group (Median: 58.13 vs. 79.17 µg/L, *P* = 0.002). This Selenium deficiency was accompanied by a marked elevation in inflammatory cytokines (TNF-α, IL-6) and oxidative stress markers (MDA), as well as a reduction in antioxidant SOD activity (all *P* < 0.001). Although cases seemed younger than controls (53.07 vs. 65.60 years, *P* < 0.001), they presented with worse metabolic profiles (lower lipid levels). After adjusting for age and gender using multivariable logistic regression, lower serum selenium levels remained significantly associated with the presence of HF, indicating that the observed deficiency may not be solely attributable to demographic differences.

**Table 1:** Baseline characteristics of included participants.

Characteristics	HF Group (*n* = 46)	Control Group (*n* = 43)	*P*-Value
Demographics			
Gender (Male), *n* (%)	21 (45.7%)	28 (65.1%)	0.065
Age (years, mean ± SD)	53.07 ± 18.90	65.60 ± 8.85	<0.001*
Selenium Status			
Selenium [µg/L, median (IQR)]	58.13 [44.13, 95.45]	79.17 [66.68, 99.27]	0.002*
Inflammatory Markers			
TNF-α (pg/mL, mean ± SD)	77.89 ± 15.62	41.99 ± 10.02	<0.001*
IL-6 (pg/mL, mean ± SD)	48.71 ± 7.52	36.98 ± 6.84	<0.001*
Oxidative Stress Markers			
MDA [nmol/mL, median (IQR)]	57.99 [51.71, 70.56]	35.82 [30.34, 40.12]	<0.001*
SOD [U/mg, median (IQR)]	0.73 [0.54, 14.26]	23.66 [17.74, 27.50]	<0.001*
Metabolic Profile			
Total Cholesterol (mmol/L)	4.45 [3.67, 4.88]	5.63 [5.34, 6.30]	<0.001*
Triglycerides (mmol/L)	1.00 [0.82, 1.25]	2.45 [2.20, 2.81]	<0.001*
LDL-C (mmol/L)	2.64 [2.15, 3.11]	3.52 [3.21, 3.97]	<0.001*
Creatinine (µmol/L)	73.60 [60.50, 94.00]	56.80 [46.02, 69.88]	<0.001*
Uric Acid (µmol/L)	382.52 ± 96.09	303.04 ± 99.16	<0.001*
NLR (mean, SD)	3.61, 2.86	2.15, 0.92	0.001769

SD, standard deviation; SOD, Serum superoxide dismutase; TSH, thyroid stimulating hormone; UA, uric acid; NLR, MDA.

* Refers to the significant *P* value < 0.05.

### 3.2 Relationship between selenium and inflammation markers

To explore the potential regulatory role of Selenium in heart failure, we performed a correlation analysis within the HF group (*n* = 46). As presented in Table 2, serum Selenium levels were significantly inversely correlated with IL-6 (r = −0.352, *P* = 0.016) and the oxidative stress marker MDA (r = −0.328, *P* = 0.026). These findings suggest that lower Selenium status may be associated with heightened inflammatory and oxidative burden in HF patients. This is consistent with Selenium's antioxidant property; a positive correlation was observed between serum Selenium and SOD activity (r = 0.455, *P* = 0.002). However, no statistically significant correlation was found between Selenium and TNF-α (r = −0.085, *P* = 0.576) in this cohort.

**Table 2:** Spearman correlation analysis between serum selenium levels and biomarkers in HF patients .

Parameters	r	*P* value
TNF-a (pg/mL)	−0.085	0.576
IL-6 (pg/mL)	−0.352	0.016*
MDA (nmol/mL)	−0.328	0.007*
SOD (U/mg)	0.455	0.002*
NLR	−0.394	0.007*

r: Spearman09 correlation coefficient. * Indicates statistical significance (*P* < 0.05). Analysis was performed on the HF group data.

### 3.3 LVEF-based subgroup comparisons

Heart failure patients were categorized by LVEF: 21 with HFpEF (LVEF ≥50%), 6 with HFmrEF (40%–49%), 11 with HFrEF (30%–39%), and 5 with Severe HFrEF (<30%). Biomarker analysis showed variations in inflammation and oxidative stress by severity, with borderline significance, *P* = 0.049. serum Selenium levels were highest in HFpEF (98.4 µg/L) and lowest in HFmrEF (59.1), increasing in HFrEF (78.7) and severe HFrEF (89.2). IL-6 levels rose from 44.3 pg/mL in HFpEF to 50.8 HFrEF but decreased to 40.8 in severe HFrEF. TNF-α peaked at 78.7 in HFrEF, while MDA (highest at 65.2 in HFrEF) indicated increased oxidative stress. SOD was most pronounced in HFrEF (11.2), reflecting a compensatory antioxidant response. These findings highlight the impact of inflammation and oxidative stress in heart failure progression.

### 3.4 Correlation heatmap analysis

**Figure 2:**
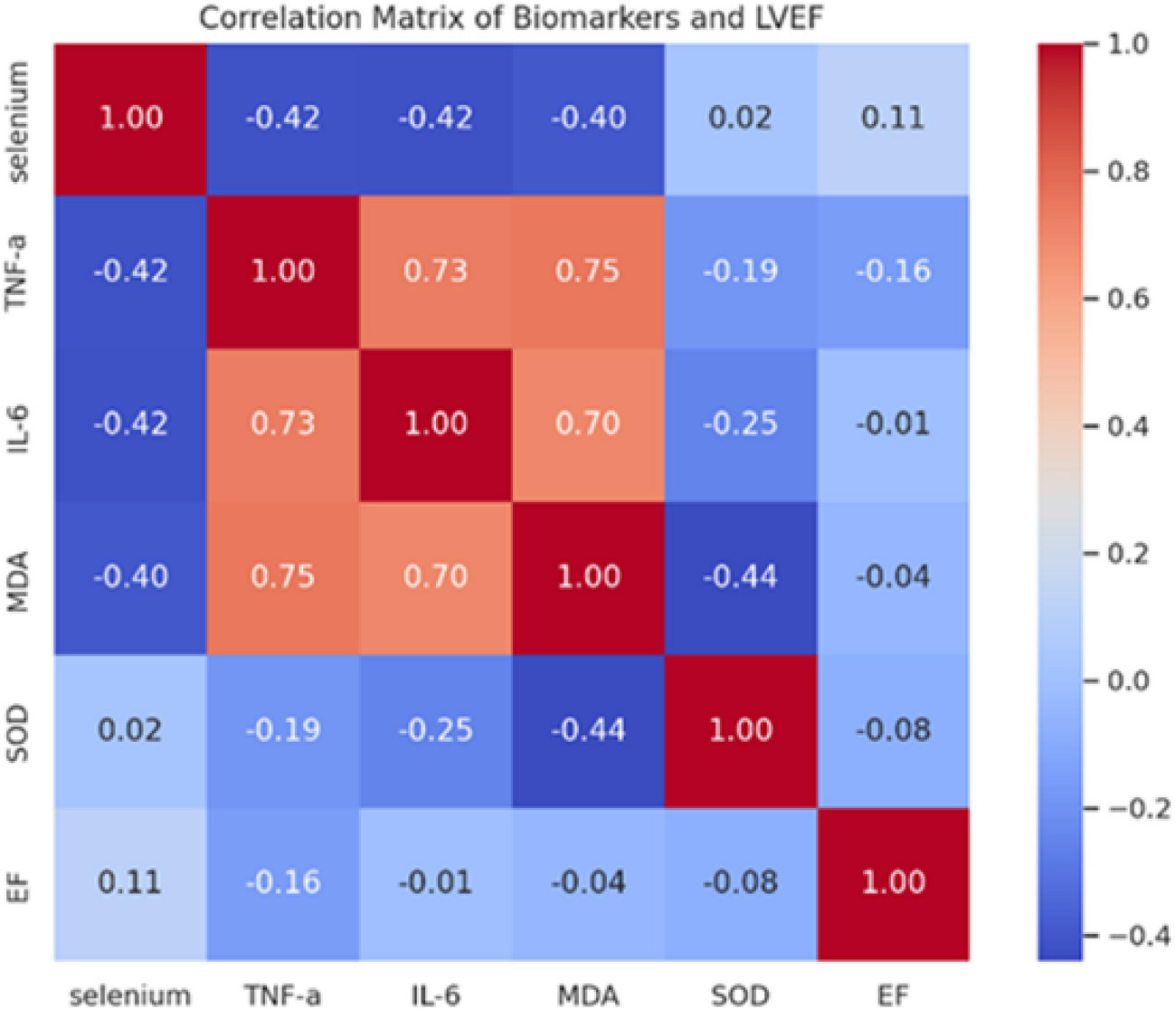
Heatmap showing correlations between Selenium, inflammatory/oxidative markers, and left ventricular ejection fraction (LVEF). Correlation heatmap of serum Selenium, inflammatory markers, oxidative stress markers, and left ventricular ejection fraction in patients with post-infarction heart failure. The heatmap shows Spearman correlation coefficients among serum Selenium, TNF-a, IL-6, MDA, SOD, and LVEF in the heart failure group (*n* = 46). Warmer colors indicate positive correlations, and cooler colors indicate negative correlations. A two-tailed *p* < 0.05 was considered statistically significant. Abbreviations: MDA, malondialdehyde; SOD, superoxide dismutase; LVEF, left ventricular ejection fraction.

To assess the interdependencies between clinical biomarkers and cardiac function, a correlation matrix was computed and visualized (Figure 2 and Table 3).

- LVEF negatively correlated with TNF-α (r = –0.27) and MDA (r = –0.36), indicating a potential role of inflammation and oxidative stress in declining cardiac function.
- Selenium exhibited a weak positive correlation with LVEF (r =  + 0.17), and negative correlation with TNF-α (r = –0.29), supporting its anti-inflammatory role.
- TNF-α and IL-6 were moderately correlated (r =  + 0.35), suggesting shared inflammatory pathways.
- The correlation between MDA and TNF-α (r =  + 0.29) also supports the interplay between oxidative stress and inflammation in HF.

These findings suggest that inflammation and oxidative stress markers are moderately interrelated and associated with cardiac function decline.

**Table 3:** Correlation between markers and LVEF.

Variable Pair	Correlation (r)	Interpretation
Selenium vs. EF	**+0**.**17**	Weak positive relationship — higher Selenium may slightly associate with better cardiac function, but not strongly.
IL-6 vs. EF	**+0**.**14**	Also, a weak positive — counterintuitive, as IL-6 is inflammatory. May reflect confounding or small sample size.
TNF-α vs. EF	**−0**.**27**	Moderate negative correlation — higher TNF-α is associated with worse cardiac function (lower EF), consistent with inflammation-driven HF.
MDA vs. EF	**−0**.**36**	Moderate negative — oxidative stress (via MDA) is meaningfully associated with lower EF, a well-known mechanism in HF pathogenesis.
SOD vs. EF	**+0**.**05**	Minimal correlation — weak relationship between antioxidant enzyme levels and EF in this dataset.
IL-6 vs. TNF-α	**+0**.**35**	Moderate positive — both cytokines are part of the same inflammator*y* axis.
MDA vs. TNF-α	**+0**.**29**	Oxidative stress is moderately linked to inflammation.
Selenium vs. TNF-α	**−0**.**29**	Higher Selenium levels are associated with **lower TNF-α**, supporting Selenium's anti-inflammatory role.

EF negatively correlates with TNF-α and MDA, supporting the hypothesis that inflammation and oxidative stress impair heart function. Selenium shows an inverse relationship with inflammation (TNF-α) but only weakly correlates with EF, suggesting it may act indirectly by modulating inflammation. The correlations are not strong enough to infer causality, but they are directionally consistent with known pathophysiology.

### 3.5 *I**n vitro* mechanistic findings

**Figure 3:**
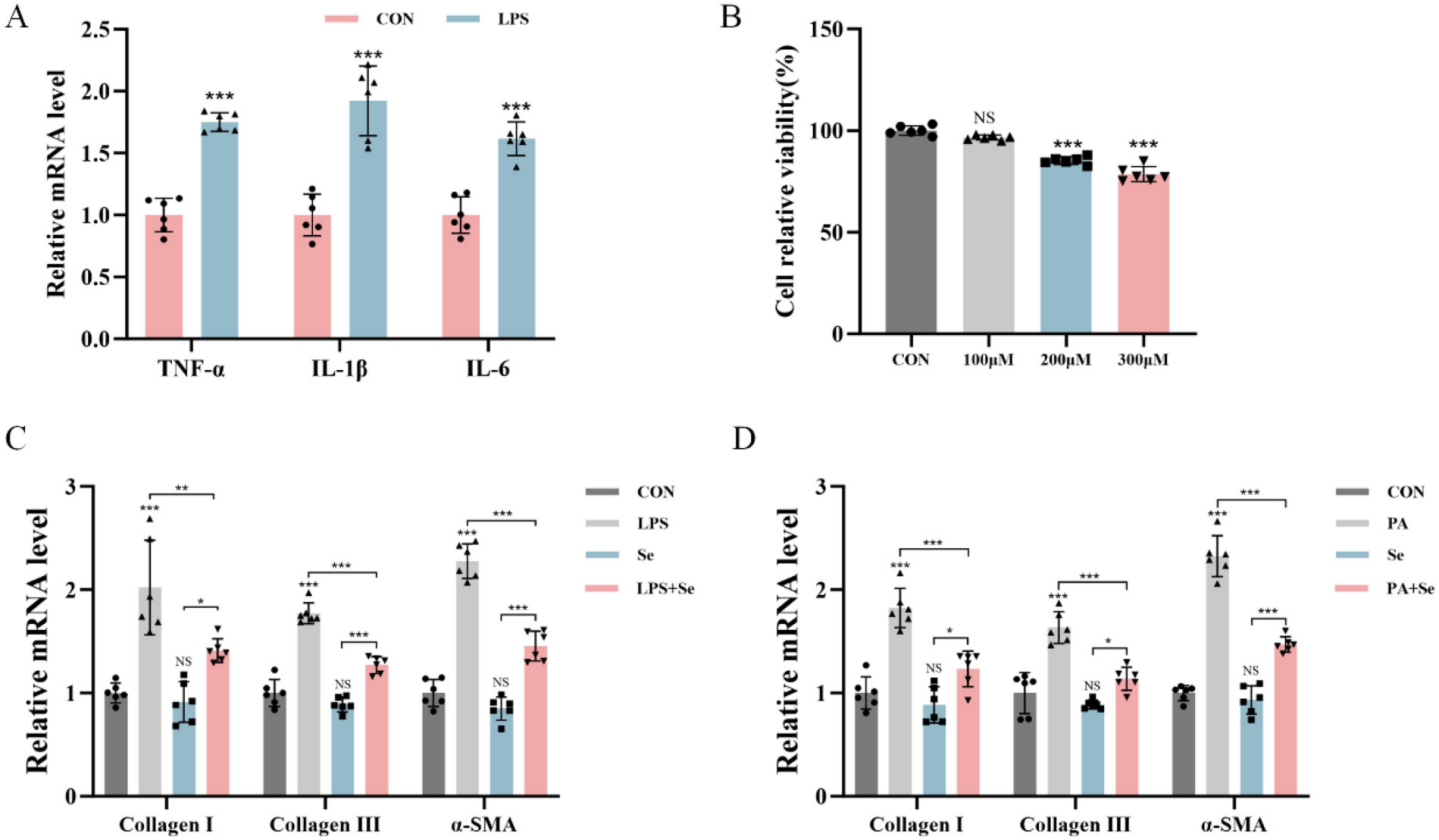
Selenium attenuates LPS- or PA-induced inflammatory response and fibrotic markers in H9C2 cells. **(A)**LPS stimulation increases selenium pro−inflammatory gene expression in H9C2 cardiomyocytes. Relative mRNA expression levels of TNF−a and IL−1b were measured by RT−qPCR in control and LPS−treated H9C2 cells. Data are presented as mean + − SD from three independent biological replicates. Statistical comparisons were performed using the unpaired Student t−test. A two−tailed *p* < 0.05 was considered statistically significant. ** *p* < 0.01 vs. control. **(B)** Effects of Selenium concentration on H9C2 cell viability. H9C2 cells were treated with sodium selenite at 100, 200, or 300 uM, and cell viability was assessed by CCK−8 assay. Data are presented as mean + − SD from three independent biological replicates. Statistical analysis was performed using one−way ANOVA followed by Dunnett multiple−comparison testing with the control group as reference. A two−tailed *p* < 0.05 was considered statistically significant. NS, not significant; ** *p* < 0.01; *** *p* < 0.001 vs. control. **(C)** Selenium attenuates LPS−induced pro−fibrotic gene expression in H9C2 cardiomyocytes. Relative mRNA expression levels of Collagen I, Collagen III, and a−SMA were measured by RT−qPCR in control cells, LPS−stimulated cells, and LPS−stimulated cells co−treated with sodium selenite (Selenium). Data are shown as mean + − SD from three independent biological replicates. Statistical analysis was performed using one−way ANOVA followed by Tukey multiple−comparison testing. A two−tailed *p* < 0.05 was considered statistically significant. NS, not significant; * *p* < 0.05; ** *p* < 0.01; *** *p* < 0.001 for the indicated comparisons. **(D)** Selenium attenuates palmitic acid−induced pro−fibrotic gene expression in H9C2 cardiomyocytes. Relative mRNA expression levels of Collagen I, Collagen III, and a−SMA were measured by RT−qPCR in control cells, PA−treated cells, and PA−treated cells co−treated with sodium selenite (Selenium). Data are shown as mean + − SD from three independent biological replicates. Statistical analysis was performed using one−way ANOVA followed by Tukey multiple−comparison testing. A two−tailed *p* < 0.05 was considered statistically significant. NS, not significant; * *p* < 0.05; ** *p* < 0.01; *** *p* < 0.001 for the indicated comparisons.

To explore the potential mechanistic link between Selenium and cardiac remodeling, an important landmark in heart failure, we performed *in vitro* experiments. Firstly, RT-qPCR results showed that LPS stimulation significantly upregulated the mRNA levels of pro-inflammatory cytokines, including TNF-α, IL-1β, and IL-6 (Figure 3A). We subsequently assessed the biological safety of Selenium using CCK-8 assays. While 100 µM Selenium had no discernible effect on H9C2 cell viability, 200 µM Selenium resulted in a slight but acceptable reduction, remaining within a robust range suitable for intervention studies (Figure 3B). Consequently, 200 µM Selenium was selected for subsequent experiments to ensure optimal biological activity. Crucially, to address whether Selenium affects basal cellular functions, a Selenium-alone group was included. Results indicated that Selenium alone did not alter the basal expression of fibrotic markers, including Collagen I, Collagen III, and α-SMA (Figures 3C,D). However, in cells challenged with LPS or PA, Selenium treatment significantly attenuated the mRNA levels of these fibrotic markers (Figures 3C,D). These findings suggest that Selenium effectively mitigates stimulant-induced myofibroblast activation without exhibiting intrinsic pro-fibrotic properties or cytotoxicity.

(A) Relative mRNA expression levels of pro-inflammatory cytokines (TNF-α, IL-1β and IL-6) in H9C2 cells treated with or without LPS for 24 h (*n* = 6 independent biological replicates). (B) Cell viability of H9C2 cells treated with various concentrations of Selenium (0, 100, 200, and 300 µM) for 24 h as measured by CCK-8 assay(*n* = 6 independent biological replicates). (C–D) Relative mRNA expression levels of fibrotic markers (Collagen I, Collagen III, and *α*-SMA) in H9C2 cells challenged with LPS or PA in the presence or absence of Selenium(*n* = 6 independent biological replicates). *P < 0.05, **P < 0.01, ***P < 0.001. Data are presented as the mean ± SD. One-way ANOVA followed by Tukey's *post-hoc* test was used for comparison (B–D).

## 4 Discussion

This combined case-control and *in vitro* study investigated the relationship between serum Selenium and markers of inflammation and oxidative stress in post-MI heart failure (HF). Post-MI HF patients on guideline-directed therapy exhibited significantly lower serum Selenium than healthy controls. Serum Selenium correlated inversely with pro-inflammatory cytokines (TNF-α, IL-6) and malondialdehyde (MDA), indicating that lower Selenium status is associated with heightened inflammatory and oxidative burden. TNF-α and MDA were positively correlated and both negatively associated with LVEF, suggesting inflammation-driven oxidative damage contributes to impaired cardiac function (7–9).

*In vitro* experiments supported the selenium clinical findings. Selenium supplementation downregulated pro-inflammatory (TNF-α, *IL-1β*) and pro-fibrotic (Collagen I, Collagen III, *α*-SMA) gene expression in cardiac cells exposed to LPS and palmitic acid. This indicates Selenium may attenuate maladaptive signaling pathways driving HF progression, providing a mechanistic complement to the correlative clinical data (7, 10, 11).

Low Selenium status is common in HF and linked to worse outcomes and reduced functional capacity (7, 12). As a component of selenoproteins essential for redox homeostasis, Selenium insufficiency exacerbates oxidative stress and inflammation in cardiac tissue (13–15). Supplementation has been shown to reduce oxidative markers, enhance antioxidant defenses, and modulate inflammatory pathways (9, 16), potentially via NF-κB signaling (11, 17).

The *in vitro* data address limitations of the observational design by demonstrating that Selenium can partially rescue stress-induced transcriptional changes in cardiomyocytes. Subgroup analysis of Seleniums revealed nonlinear associations (see Supplementary Figure S1): patients with HFpEF and severe HFrEF had higher Selenium, while those with intermediate HFrEF showed the lowest Selenium and highest inflammatory/oxidative profiles. Elevated SOD activity in HFrEF may reflect compensatory antioxidant upregulation before depletion as disease advances (18).

Mixed cardiovascular outcomes in Selenium trials underscore the importance of baseline selenium status. Trials in Selenium-replete populations showed no benefit (19), whereas supplementation in deficient or older cohorts improved cardiovascular mortality and biomarkers (20, 21).

Limitations include the cross-sectional design, age differences between groups, used of a rat-derived H9C2 cell line, and reliance on mRNA data without protein validation. Future studies should employ age-matched, prospective cohorts and confirm findings in primary human cells.

In summary, our findings support an association between low Selenium and elevated inflammatory/oxidative burden in post-MI HF and provide a mechanistic rationale for Selenium's protective role against inflammation- and fibrosis-driven dysfunction. Further prospective studies are needed to define Selenium's therapeutic potential in HF.

## 5 Conclusions

Lower serum selenium was associated with greater inflammatory and oxidative stress burden in patients with post-myocardial infarction heart failure. The *in vitro* experiments further suggested that Selenium may modulate inflammation- and fibrosis-related pathways under stress conditions. Together, these findings support serum Selenium as a candidate biomarker for further study in heart failure, while prospective and interventional studies are needed to determine clinical relevance and causality.

## Data Availability

The raw data supporting the conclusions of this article will be made available by the authors, without undue reservation.

## Supplementary material

Supplementary Figure S1 Serum selenium levels across LVEF-based heart failure phenotypes. Serum selenium concentrations are shown across four left ventricular ejection fraction (LVEF)−defined heart failure subgroups: HFpEF (LVEF ≥50%), HFmrEF (40%–49%), HFrEF (30%–39%), and severe HFrEF (<30%). Box−and−whisker plots display the distribution of selenium levels within each subgroup. The central line represents the median, boxes indicate the interquartile range (IQR), and whiskers extend to 1.5× IQR. Individual data points are overlaid to illustrate sample distribution. Dots: Individual patient serum selenium values; Boxes: Interquartile range (IQR); Horizontal line in box: Median Whiskers: 1.5 × IQR; Dashed curve: LOESS−smoothed trend indicating non−linear distribution; n: Number of participants in each subgroup
