# Supplementary material for: Association of serum selenium levels with inflammatory and oxidative stress markers in patients with post-infarction heart failure: an exploratory case-control study supported by in vitro mechanistic evidence
Source: Front Cardiovasc Med. 2026 Jun 9;13:1821567. doi: 10.3389/fcvm.2026.1821567 (PMC13288326; doi:10.3389/fcvm.2026.1821567)
Supplement: Supplementary Figure S1 — Serum selenium levels across LVEF-based heart failure phenotypes. Serum selenium concentrations are shown across four left ventricular ejection fraction (LVEF)−defined heart failure subgroups: HFpEF (LVEF ≥50%), HFmrEF (40%–49%), HFrEF (30%–39%), and severe HFrEF (<30%). Box−and−whisker plots display the distribution of selenium levels within each subgroup. The central line represents the median, boxes indicate the interquartile range (IQR), and whiskers extend to 1.5× IQR. Individual data points are overlaid to illustrate sample distribution. Dots: Individual patient serum selenium values; Boxes: Interquartile range (IQR); Horizontal line in box: Median Whiskers: 1.5 × IQR; Dashed curve: LOESS−smoothed trend indicating non−linear distribution; n: Number of participants in each subgroup [file Image1.pdf]

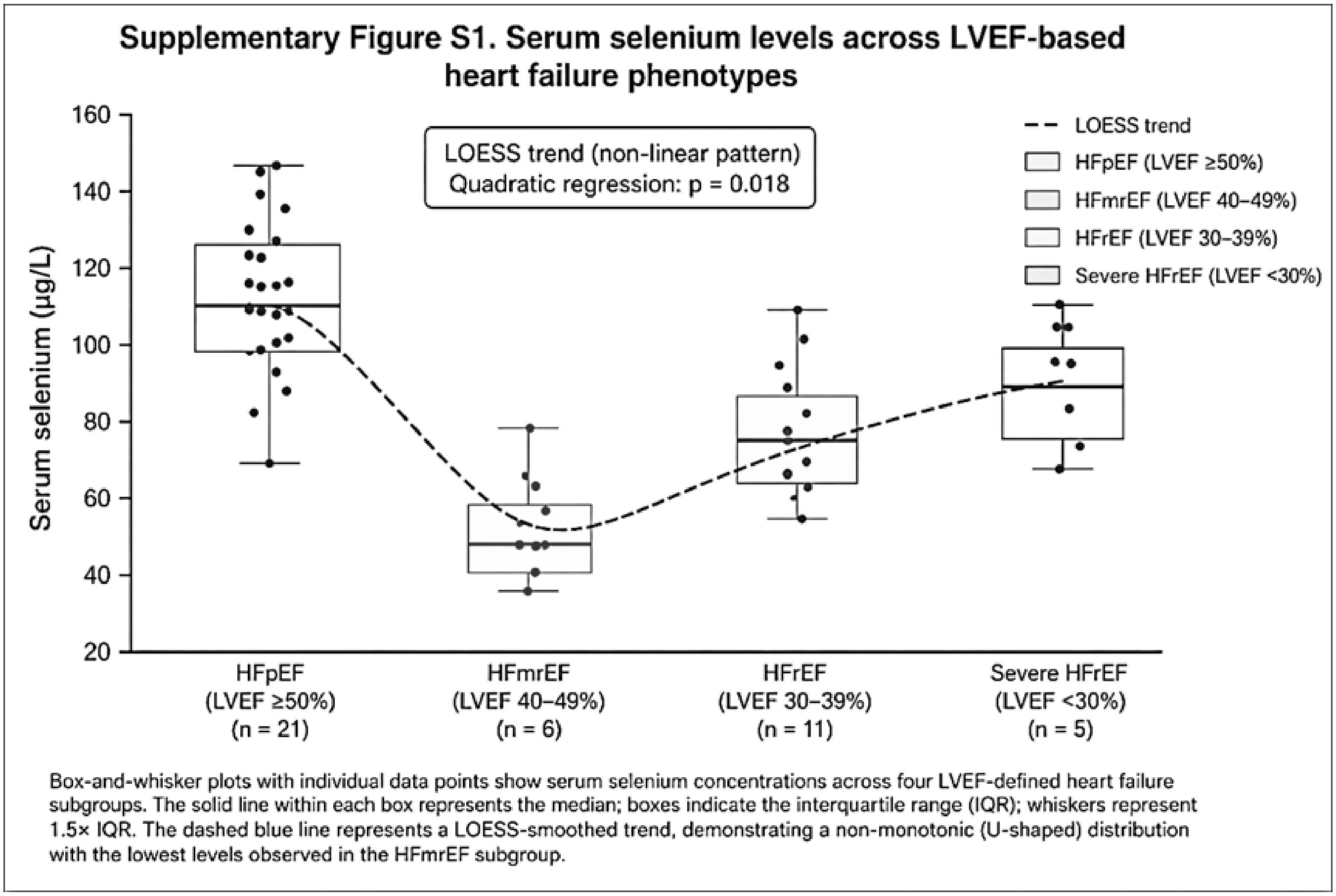

Figure S1. Serum selenium levels across LVEF-based heart failure phenotypes.

Serum selenium concentrations are shown across four left ventricular ejection fraction (LVEF)-defined heart failure subgroups: HFpEF (LVEF ≥50%), HFmrEF (LVEF 40%–49%), HFrEF (LVEF 30%–39%), and severe HFrEF (LVEF <30%). Box-and-whisker plots display the distribution of selenium levels within each subgroup. The central line represents the median, boxes indicate the interquartile range (IQR), and whiskers extend to 1.5 × IQR. Individual data points are overlaid to illustrate sample distribution. Dots: individual patient serum selenium values; boxes: interquartile range (IQR); horizontal line in box: median; whiskers: 1.5 × IQR; dashed curve: LOESS-smoothed trend indicating non-linear distribution; n: number of participants in each subgroup.
